# Further Observations on Diphtheria

**Published:** 1878-05

**Authors:** 


Further Observations on Diphtheria.—Since my previous communication regarding diphtheria, written 4th January last, the disease has continued to prevail, although not so extensively. I have attended a number of cases, some of them severe. The same treatment has been pursued, and with uniform success. I have seen three cases which proved fatal; in two of them I was called in as consulting physician; one died of an affection of the trachea, for whom nothing could have been done; the other died of septicæmic poisoning and hemorrhage. The other case had been ill over a week; nothing had been done for five days. The throat was filled with a fœtid, black, putrid mass, with a brown fluid flowing from the mouth and nostrils. Next day hemorrhage set in, which soon ended fatally. Death in this case was evidently caused by the membrane becoming putrid. The soft parts beneath became also affected, ending in fatal hemorrhage.

I have learned a number of facts regarding diphtheria which are very important: First, that the disease is always caused by a specific contagion, spreading from one person to another, either by personal contact or from articles of clothing, in a similar way to scarlatina and other zymotic diseases. That it does not arise from dirty cesspools, rotten carpets, etc., although I am willing to admit that individuals whose blood is filled with emanations from these substances will be more likely to be affected by the contagion, and have the disease in greater severity. The period of incubation varies from seven to ten days. Certain general symptoms, such as chill, headache, etc., always precede the sore throat. I have seen several cases which have been exposed to diphtheria here, furnish symptoms lasting one or two days without very sore throat, but who seem capable of giving true diphtheria to others.

I saw one man aged 50, who had all the symptoms without any sore throat, and who had been in close attendance upon a grand child who died. A week after his slight illness a son of his had an attack, and a young man aged 16 at the same time had a severe attack, and he had been in the habit of associating with the old man, one night lying in bed with him, and smoking from the same pipe. Many have diphtheria so slightly that they are not aware that much is the matter with them, and continue their avocations, thus unwittingly spreading the disease. This is more particularly the case with children attending school. During the past six months there have been over 200 cases in this place, and in not one has the disease been known to return, at least so far as the other medical gentleman and myself know, and I have been making particular enquiries. I therefore feel confident, that like scarlatina, etc., the disease is not likely to return to the same person for at least many years.

I will relate what happened in one family, which shows clearly the protection those possess who have recently gone through the disease. In the month of October last, four little children, members of one family, had each diphtheria, a few days intervening between each case. Their mother, a lady of about 40 years of age, attended them at the time, swabbing their throats regularly. They all recovered, their mother remaining unaffected. Three months afterwards they visited a family who had a girl, aged 11, very ill of diphtheria. In about ten days she had a very severe attack, apparently caught from visiting the sick child. The children were in the room with her constantly, having no one else to look after them. It is nearly a month since Mrs. W. recovered, and the children still continue in perfect health, and have shown no symptom of disease. I could relate several cases of nearly a similar character. Many escape the disease, although fully exposed, and they catch it from a very slight exposure. One of our doctors here had been visiting patients all winter and remained well, and once when swabbing a throat the patient coughed, and a piece of membrane flew into his mouth, but he still remained unaffected. Lately he made a friendly visit to a young man, about his own age, and who had a pretty severe attack. A week afterwards he was seized with diphtheria in its usual form.

In my former article I neglected to give what I believe to be the rationale of the treatment. The swabbing material, consisting of No. 2 tinct. of iron, sulphurous acid, carbolic acid and glycerine, destroys the morbid matter contained in the false membrane, and also prevents its decomposition. Its frequent application is necessary to keep up its antiseptic effect, as it must be washed off by the swallowing of liquids. Next, the internal medicine, consisting of chlorat. potass. tinct. ferri, mur. and glycerine, and water taken between the periods of swabbing, acts as an antiseptic, but being swallowed it is absorbed and kills the morbid matter contained in the blood, and by being continued even after the recovery, prevents the sequelæ which sometimes follow diphtheria. The cold water application I consider of great importance. It keeps down congestion, and prevents the further formation of the membrane, and also favors its separation. When diphtheria breaks out in a family, I make all the other members use sulphurous acid freely, as I believe it has a powerful effect as a preventive, by killing the contagion as it enters the body. I also direct the rooms to be fumigated regularly by burning sulphur on hot cinders. This precaution I constantly recommend, as I believe it tends to prevent the spreading of the disease.—*Canada Medical and Surgical Journal.*

